# 

**Published:** 1903-11-14

**Authors:** 


### CONTENTS.

**The Crisis at St. Bartholomew's Hospital** - 109

**The Food of the People** - 110

**Annotations** - 111

**Notes and News** - 112

**Hospital Clinics and Medical Progress—**

Dr. Otto Schmidt's Specific Treatment of Cancer - 113

The Treatment of Uterine Fibroids - 113

Congenital Hypertrophic Stenosis of the Pylorus - 114

Cancer of the Prostate - 115

Progress in Psychiatry - 116

Progress in Neurology - 117

Respiratory Diseases - 118

**New Appliances and Things Medical** - 119

**The Book World of Medicine and Science** - 120

**Hospital Administration—**

The Future of St. Bartholomew's Hospital - 121

Visits to Private Asylums - 123

**Editor's Letter-Box** - 123

**Scraps and Gleanings** - 124

**The Student of Medicine** - 124

*NURSING SECTION.*

**Notes on News from the Nursing World—** Imperial Military Nursing Service—Queen's Nurses for Ireland—The Nurses of the Rotunda Hospital and their Master—Hospital-Trained Nurses and Poor-Law Appointments—Male Nurses at the Poplar Sick Asylum—Gross Carelessness at Dudley Hospital—The Training of Nuns as Nurses—"Nazarene Nurses"—Unfounded Charges Against an Infirmary Nurse—Gratuity to the Matron of a Workhouse Infirmary—Zenana Bible and Medical Mission—The District Nursing Question at Cheltenham—The Dietary at Newton Abbot—Concerts for Nursing Associations—Our Christmas Distributions—Short Items - 87-90
**Some Practical Points in the Nursing of Diseases of the Skin** - 91
**The Prevention of Bed-sores** - 92
**Workhouse Nursing** - 93
**Central Midwives Board** - 94
**New Books on Nursing** - 95
**Presentations** - 95
**Official Announcements** - 95
**Appointments** - 96
**Everybody's Opinion** - 96
**Echoes from the Outside World** - 97
**For Reading to the Sick** - 98
**Notes and Queries** - 98

